# Molecular Subtyping to Detect Human Listeriosis Clusters

**DOI:** 10.3201/eid0906.020702

**Published:** 2003-06

**Authors:** Brian D. Sauders, Esther D. Fortes, Dale L. Morse, Nellie Dumas, Julia A. Kiehlbauch, Ynte Schukken, Jonathan R. Hibbs, Martin Wiedmann

**Affiliations:** * Wadsworth Center, New York State Department of Health, Albany, New York, USA; † Cornell University, Ithaca, New York, USA; ‡ University at Albany, Albany, New York, USA

**Keywords:** *Listeria monocytogenes*, foodborne, outbreak, detection, clustering analysis, pulsed-field gel electrophoresis, PFGE, ribotype, surveillance, research

## Abstract

We analyzed the diversity (Simpson’s Index, D) and distribution of *Listeria monocytogenes* in human listeriosis cases in New York State (excluding New York City) from November 1996 to June 2000 by using automated ribotyping and pulsed-field gel electrophoresis (PFGE). We applied a scan statistic (p<0.05) to detect listeriosis clusters caused by a specific *Listeria monocytogenes* subtype. Of 131 human isolates, 34 (D=0.923) ribotypes and 74 (D=0.975) PFGE types were found. Nine (31% of cases) clusters were identified by ribotype or PFGE; five (18% of cases) clusters were identified by using both methods. Two of the nine clusters (13% of cases) identified corresponded with investigated multistate listeriosis outbreaks. While most human listeriosis cases are considered sporadic, highly discriminatory molecular subtyping approaches thus indicated that 13% to 31% of cases reported in New York State may represent single-source clusters. Listeriosis control and reduction efforts should include broad-based subtyping of human isolates and consider that a large number of cases may represent outbreaks.

*Listeria monocytogenes* is a bacterial foodborne pathogen that can cause severe invasive disease manifestations, including abortion, septicemia, and meningitis. While multiple large outbreaks have been recognized, most cases are thought to be sporadic (*1*). Human listeriosis is relatively rare, typically includes long incubation periods (7–60 days), usually results in hospitalization (85% to 90%), and often results in death (<30%) (*2*). Persons with specific immunocompromising conditions, pregnant women, and newborns appear to be particularly susceptible to invasive listeriosis, and most reported cases occur in these specific risk groups (*3*,*4*). Various studies indicate that from 1% to 5% of common ready-to-eat foods may contain *L. monocytogenes* (*5*–*7*), and these foods may be widely distributed as a result of current marketing and distribution practices. Traditional epidemiologic surveillance alone may not detect many common source outbreaks, particularly if a limited number of cases occur over a wide geographic area (*8*,*9*) because of the unique characteristics of human foodborne listeriosis.

Subtyping methods for *L. monocytogenes* include phenotypic (e.g., serotyping and phage-typing) as well as different DNA-based subtyping methods. Phenotypic methods often yield a low power of discrimination in strains (e.g., >90% of all human isolates represent 3 of the 13 known serotypes), suffer from biologic variability (e.g., phage typing), and may not be applicable to all strains (*10*). Molecular subtyping methods include multilocus enzyme electrophoresis, ribotyping, pulsed-field gel electrophoresis (PFGE), polymerase chain reaction (PCR), and restriction-fragment length polymorphism (RFLP) analysis. Automated ribotyping was previously used for rapid subtyping *L*. *monocytogenes* for source tracking, population genetics–based studies, and epidemiologic investigations (*11*–*13*); however, it is expensive and not as discriminatory as PFGE (*14*). PFGE provides sensitive subtype discrimination and is often considered the standard subtyping method for *L. monocytogenes* (*15*). However, this method is not automated and is labor intensive. Even recently developed rapid protocols take approximately 30 hours to perform (*10*,*15*).

We used two molecular subtyping methods (automated *Eco*RI ribotyping and *Asc*I PFGE) to evaluate and compare their discriminatory power and utility and to estimate the incidence of single source clusters among human listeriosis cases. A scan statistic with an underlying Poisson distribution was used to detect the occurrence of temporal clusters caused by indistinguishable subtypes. A space-time scan statistic was used to evaluate spatial and temporal clustering on the basis of county of patient residence and a 3-month window.

## Materials and Methods

### Isolates and Case Reporting

In New York State, Public Health Law 2102 requires that laboratories and physicians immediately report isolation of *L. monocytogenes* from a sterile site (e.g., blood or cerebrospinal fluid) to public health authorities (*16*). Furthermore, local diagnostic and clinical laboratories are asked to submit all *L. monocytogenes* isolates to the New York State Department of Health Wadsworth Center. Through this system, *L. monocytogenes* isolates from cases of human invasive disease among New York State residents (excluding New York City, which is served more directly by the local health department) were collected over 44 months (November 1996 through June 2000). Only one isolate per patient was analyzed; therefore, each isolate in this study represents a single, unique listeriosis case. All isolates were confirmed by conventional biochemical tests at the Wadsworth Center. Standardized *L. monocytogenes* serotyping reagents were not available and serotyping was thus not performed.

County health departments reported epidemiologic information to the New York State Department of Health’s Bureau of Communicable Disease. Local health department’s systematic review of case reports aided identification of potential outbreak cases when large increases in listeriosis cases (irrespective of subtype) were reported. Our study is a retrospective laboratory subtype analysis, which did not include routine comprehensive risk factor analysis (i.e., history of food eaten).

### Automated Ribotyping

Ribotyping was performed by using the restriction enzyme *Eco*RI and the RiboPrinter Microbial Characterization System (Qualicon Inc., Wilmington, DE) as previously described (*17**,**18*).

### PFGE Analysis

PFGE was performed according to PulseNet protocol (*15*). *Apa*I PFGE patterns typically display more bands than *Asc*I patterns and may offer higher levels of discrimination; however, *Asc*I patterns typically have patterns with bands that are more easily analyzed by software and the human eye because of greater average distances between bands. While the current PulseNet protocol (*15*) recommends the use of both *Apa*I and *Asc*I for PFGE typing of *L. monocytogenes,* only *Asc*I was used in this study, which was initiated before formal inclusion of *L. monocytogenes* into PulseNet. Bacterial cultures were embedded in agarose, lysed, washed, and digested with the restriction enzyme *Asc*I for 4 h at 37°C and electrophoresed on a Chef Mapper XA (BioRad Laboratories, Hercules, CA) at 6 V/cm for 22 h with switch times of 4 s to 40.01 s. Pattern images were acquired by using a BioRad Gel Doc with Multi Analyst software (Bio-Rad Laboratories) (v. 1.1) and compared by using the Applied Maths Bionumerics (Applied Maths, Saint-Martins-Latem, Belgium) (v. 2.5) software package. Pattern clustering was performed by using the unweighted pairs group matching algorithm and the Dice correlation coefficient (*15*).

### Strain Nomenclature

Ribotype patterns were automatically assigned a DuPont ID (e.g., DUP-1044) by the Riboprinter Microbial Characterization System (Qualicon, Inc.); each pattern was confirmed by visual inspection. If visual inspection found that a given DuPont ID included more than one distinct ribotype pattern, each pattern was designated by an alphabetically assigned additional letter (e.g., DUP-1044A and DUP-1044B represent two distinct ribotype patterns within DuPont ID DUP-1044). Distinct ribotype patterns within a given DuPont ID generally differed by position of a single weak band. If a ribotype pattern did not match a DuPont ID pattern with a similarity >0.85, a type designation was assigned manually based on the ribogroup assigned by the instrument (e.g., ribogroup 116-363-S-2). Ribotype patterns (and other subtype data) for isolates in this study are available for comparison on the Internet (available from: URL: www.pathogentracker.net). PFGE patterns differing by at least one band from a previously recognized type were given an indexed type comprise a two-letter geographic prefix, a four-digit year of first isolation, a three-letter restriction enzyme code, and a four-digit sequential number (e.g., NY1996ASC0001).

### Simpson’s Index of Discrimination

The suitability of typing methods for differentiation of strains was determined by using Simpson’s Numerical Index (*19*). This index was calculated for each typing method, as well as for the combination of both methods.

### Cluster Detection Algorithm by Using a Scan Statistic

**Figure 1:**
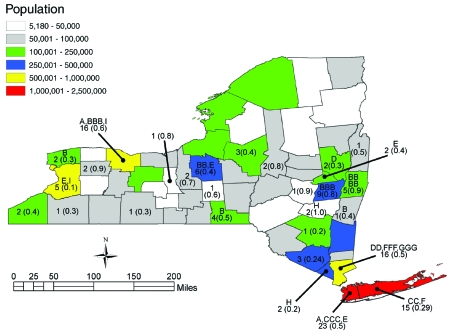
Dispersion of listeriosis cases, New York State (excluding New York City), November 1996–June 2000. Comparison of New York State population base overlaid with temporal listeriosis clusters from Table 1 (indicated by letter; defined by ribotype and pulsed-field gel electrophoresis type). Cases per county and annualized rate per 100,000 (in parentheses) are shown. New York City listeriosis data are not included in this study.

The scan statistic (*20*,*21*) maintains the assumption that an underlying Poisson distribution and a stable population at risk over time describes the occurrence of rare events. This statistic tests the null hypothesis that the incidence of events within a given time window is equal to the incidence of events outside the window. We used a conditional Poisson distribution to describe the occurrence of individual *L. monocytogenes* subtypes over 44 months. Since the incubation period of listeriosis can be up to 70 days, we determined the temporal distribution of ribotypes and PFGE types for both 1- and 3-month windows. To determine the threshold value of occurrences, indicating a larger than expected number of events per window, we compared the number of occurrences in a given period to the expected maximum number of events in a given window. The expected number is calculated under the assumption that individual occurrences occur randomly with an identical rate over time. The conditional Poisson probability is given by: P(n | N, p)= Pr(n_obs_ > n), where n_obs_ is the observed cluster size in a window, N is the total number of events during the total period, and p is the relative window length (the length of the window in months divided by the length of the total period). Statistically significant clusters were identified when the number of isolates with a given subtype in the study window was greater than expected under the Poisson assumption. Exact p values were obtained from statistical tables (*22*). Clusters of ribotypes or PFGE types were evaluated by using a Poisson distribution with a mean rate equal to the number of occurrences divided by the total observation period. No corrections for seasonality were applied because analysis was performed at the subtype level and a large number of subtypes were observed. Furthermore, the prevalence of the different subtypes did not vary consistently by season, and correction was not warranted. Time-space clustering was evaluated by using a similar algorithm (SatScan 2.1.3, National Cancer Institute 1998) (*23*); however, only 3-month windows were evaluated. Case numbers were converted to case rate (cases/100,000) to account for the source population size. Time clustering within a 3-month window was combined with space clustering in an area with a maximum space window size of 15% of the total space. Kulldorff (*24*) recommended using a maximum space window size of up to 50% of the total space, while others reported using smaller values on the basis of the geographic boundaries studied. Norstrom et al. (*25*) used a 10% space window to avoid scanning outside the geographic region of study. We used a window of 15% of the total space. Since exact patient location (zip code or address) was not available, analysis was performed by using county of residence data, which provided a better chance of identifying local clusters within a small food distribution area. Each case was assigned the spatial coordinates of the county in which the patient was residing (Figure 1). Statistical significance for all tests was defined as p<0.05.

## Results

### Cases

From November 1996 through June 2000, a total of 135 *L. monocytogenes* isolates were collected from human cases, with four mother/newborn pairs of isolates. All four isolates from the newborns matched the subtype of the respective mother and were not included in our analysis. The incidence of reported isolates ranged from 0 to 13 per month, with a median of three. Isolates were seasonally distributed with peaks in July 1997 (n=5), October 1998 (n=13), and September 1999 (n=9). On the basis of the 2000 census population estimate of 10,968,179 (*26*) for New York State (excluding New York City), we detected a listeriosis rate of 0.33 cases per 100,000. Cases were distributed across the state (Figure 1). Case-patient ages ranged from <1 day to 98 years with a median age of 66 years. Gender was reported for all cases; 76 (58%) of case-patients were female.

A total of 34 ribotypes and 74 PFGE types were differentiated among the 131 human isolates; 19 ribotypes and 50 PFGE types were unique (i.e., represented by only one patient isolate). Ribotypes DUP-1044A and DUP-1052A were each prevalent in >10% of cases, and these two ribotypes alone accounted for 39% of cases. One PFGE type (NY1997ASC0010) accounted for 13% of cases. No other PFGE types accounted for >5% of cases.

### Discriminatory Ability of Typing Methods

Simpson’s Index was used to determine the discriminatory power of the subtyping methods used. The D value was 0.923 for ribotyping, 0.975 for PFGE, and 0.980 for combined use of both typing techniques. PFGE further discriminated most ribotypes (Table 1); however, three isolates with indistinguishable PFGE types were further differentiated into two ribotypes (Table 1, cluster E).

**Table 1:** Temporal clusters of human listeriosis identified by ribotyping, pulsed-field gel electrophoresis (PFGE), or both by using a 3-month window, New York State, November 1996–June 2000^a^

					p values for temporal scan statistic with:			
Cluster	Ribotype	Ribotype	PFGE type	Date of specimen collection	*Asc*I PFGE type (no.)	PFGE relatedness	
A	DUP-1044A	NS	<0.05	**Sep 1997**	**NY1997ASC0016**	**Indistinguishable**	
				**Sep 1997**	**NY1997ASC0016**	**Indistinguishable**	
B	DUP-1044A^a^	<0.01	<0.05	**Sep 1998**	**NY1997ASC0006**	**1 band from NY1997ASC0010**	
				**Oct 1998**	**NY1997ASC0010 (7)**	**Indistinguishable**	
				**Nov 1998**	**NY1997ASC0010 (2)**	**Indistinguishable**	
				**Dec 1998**	**NY1997ASC0010 (4)**	**Indistinguishable**	
C	DUP-1042B	<0.05	N/A	Dec 1998	NY1999ASC0045	>5 bands from all others in cluster	
				**Feb 1999**	**NY1997ASC0017**	**2 bands from NY1997ASC0014**	
				Feb 1999	NY1996ASC0001	>5 bands from all others in cluster	
				Feb 1999	NY1999ASC0050	>5 bands from all others in cluster	
				**Mar 1999**	**NY1997ASC0014**	**2 bands from NY1997ASC0017**	
D	DUP-1042B	NS	<0.05	Aug 1999	NY1997ASC0017	>5 bands from all others in cluster	
				**Aug 1999**	**NY1999ASC0050**	**2 bands from NY1999ASC0061**	
				**Aug 1999**	**NY1999ASC0061**	**Indistinguishable**	
				**Sep 1999**	**NY1999ASC0061**	**Indistinguishable**	
E	116-363-S-2	<0.01	<0.01	**Aug 1999**	**NY1999ASC0052**	**Indistinguishable**	
	116-363-S-2	<0.01	<0.01	**Sep 1999**	**NY1999ASC0052**	**Indistinguishable**	
	DUP-1044B	NS	<0.01	**Sep 1999**	**NY1999ASC0052**	**Indistinguishable**	
	116-363-S-2	<0.01	NS	Sep 1999	NY1999ASC0064	4 bands from NY1999ASC0052	
F	DUP-1053A	<0.05	<0.05	**Sep 1999**	**NY1999ASC0069**	**Indistinguishable**	
				**Nov 1999**	**NY1999ASC0069 (2)**	**Indistinguishable**	
				**Dec 1999**	**NY1999ASC0069**	**Indistinguishable**	
G	DUP-1052A^a^	NS	<0.05	**Oct 1999**	**NY1997ASC0018 (3)**	**Indistinguishable**	
H	DUP-1043	<0.01	<0.05	**Jan 2000**	**NY2000ASC0075**	**Indistinguishable**	
				**Feb 2000**	**NY2000ASC0075**	**Indistinguishable**	
I	DUP-1045B	<0.05	<0.05	**Apr 2000**	**NY2000ASC0077**	**Indistinguishable**	
				**May 2000**	**NY2000ASC0077**	**Indistinguishable**	
				May 2000	NY2000ASC0083	>5 bands from NY2000ASC0077	

^a^Epidemiologically linked clusters; cluster B linked to eating hot-dog brand 1, cluster G to eating paté brand 2; bold, isolates for which PFGE patterns were supportive of respective clusters; NS, not significant (p>0.05); N/A, the statistical significance of occurrence of a unique PFGE type cannot be tested.

### Cluster Detection

**Figure 2:**
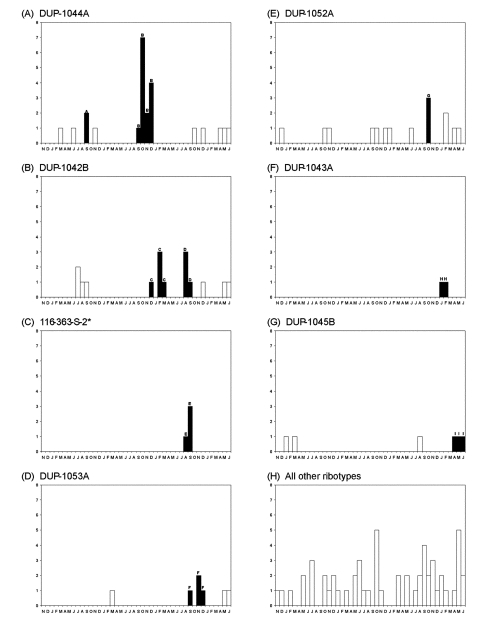
Temporal distribution of listeriosis clusters detected based on ribotype or pulsed-field gel electrophoresis (PFGE) data, using a 3-month window scan statistic. Panels A–G each show the distribution of cases caused by a specific ribotype, ribotypes are denoted in the header of each panel. For panel C, one case caused by ribotype DUP-1044B is included with cases caused by ribotype 116-363-S-2 based on a PFGE match (Table 1, cluster E). Cases, which are part of statistically significant ribotype or PFGE clusters are denoted by dark bars and labeled by cluster designation (A–I, see Table 1). Open bars indicate cases that were not part of a cluster detected by the scan statistics. Panel H shows human cases, which did not represent clusters and were not caused by any of the ribotypes shown in panels A–G. The X-axis of each panel represents November 1996 to June 2000.

Ribotyping and PFGE subtyping data were analyzed separately by using a scan statistic on 1- and 3-month windows to detect statistically significant clusters of identical ribotypes and PFGE types. A total of 9 clusters representing 41 (31%) cases were detected by ribotyping, PFGE, or both (Tables 1 and 2). Clusters were detected throughout the study period (Figure 2). Two clusters (B and G) were epidemiologically linked to national outbreaks and known sources and included 17 (13%) cases. The remaining seven clusters were not epidemiologically defined as outbreaks, and the exact source of exposure was undetermined. Ribotype-based scanning with 1-month periods detected two clusters (Table 1, B and E), with 3-month periods, and scanning detected six clusters (B, C, E, F, H, and I). PFGE-based scanning with 1-month periods detected five clusters (A, B, E, F, G), with 3-month periods; eight clusters were detected (A, B, D, E, F, G, H, and I). All clusters identified by using 1-month periods were also identified by using 3-month periods.

**Table 2:** Comparison of statistically significant temporal listeriosis clusters stratified by subtyping technique used to detect and confirm each cluster

Clusters	Cluster definition	No. of clusters	No. of cases (%)^a^
1. Clusters detected by ribotype or PFGE	Ribotype clusters or PFGE clusters detected by using the scan statistic (p<0.05)	9	41 (31)
2. Ribotype clusters	Indistinguishable ribotype pattern clusters detected by the scan statistic (p<0.05)	6	31 (24)
2a. Ribotype clusters supported by PFGE	Ribotype clusters, containing closely related PFGE types (<3 bands difference)	6	26 (20)
3. PFGE clusters	Indistinguishable PFGE patterns detected by the scan statistic (p<0.05)	8	31 (24)
3a. PFGE clusters supported by ribotype	PFGE clusters, which contained identical ribotype patterns	8	30 (23)
4. Clusters supported by ribotype and PFGE	Clusters detected as 2a and 3a	5	23 (18)
5. Epidemiologically linked ribotype or PFGE clusters	Clusters detected by ribotype, PFGE, or both and supported by epidemiologic data	2	17 (13)

^a^Based on total sample population of 131 isolates; PFGE, pulsed-field gel electrophoresis.

**Figure 3:**
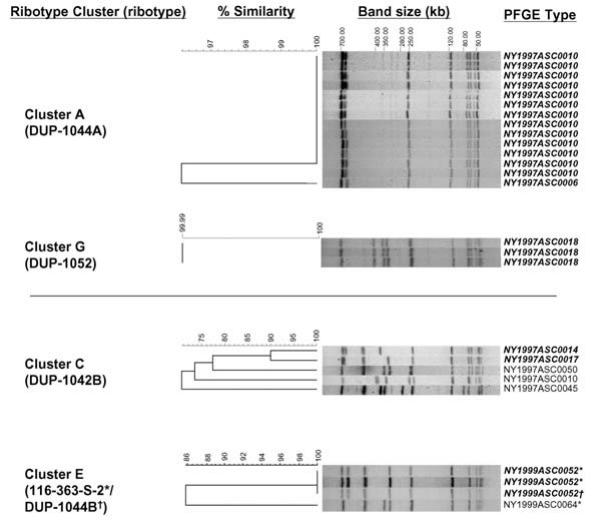
Comparison of *Asc*I pulsed-field gel electrophoresis (PFGE) patterns for isolates from selected ribotype clusters. *Asc*I PFGE types are shown for two clusters representing epidemiologically confirmed outbreaks (A and G), one ribotype cluster that was further discriminated by PFGE typing (C), and one cluster with overlapping PFGE and ribotype clusters (E). Isolates with <3 bands difference are shown in bold. The percent similarity does not reflect true phylogenetic distance.

A total of six ribotype-based clusters (Table 1; B, C, E, F, H, and I) of two or more isolates (p<0.05) were detected, representing a total of 31 (24%) cases. PFGE alone identified eight clusters (A, B, D, E, F, G, H, and I), representing a total of 31 (24%) cases. Ribotyping and PFGE results were used to further refine clusters detected by the scan statistic. All six ribotype clusters contained at least two indistinguishable or closely related (<3 bands different) PFGE patterns. For the purpose of refining ribotype clusters, we interpreted PFGE patterns differing by <3 bands from each other as possibly being clonally related and sharing a recent enough common ancestor to be grouped together for epidemiologic investigations (*27*). Three of these clusters (C, E, and I) contained one or more isolates removed from the ribotyped-based cluster because they were considered not closely related to the most common PFGE pattern in the respective cluster (see Figure 3 for two examples of ribotype clusters with multiple PFGE types and two examples of ribotype clusters with indistinguishable or closely related PFGE types). Overall, ribotype clusters that were further supported by indistinguishable or closely related PFGE types represented 26 (20%) cases (Table 2). Of the eight PFGE clusters detected, all, except one (Figure 3, cluster E), comprised isolates with identical ribotypes. Overall, five clusters (B, E, F, H, and I; 23 cases) were detected by the temporal scan statistic on the basis of both ribotype and PFGE data.

Space-time cluster analysis independently identified three of the ribotype clusters (B, G, and H) and five of the PFGE clusters (B, D, G, H, and I). While some geographic clusters were located within one county (D and G), others comprised cases in one or more counties (B, H, and I). Cluster B comprised two main geographic clusters; one included five cases (Rensselaer and Columbia Counties), and the other included six cases (Broome, Monroe, and Onondaga Counties). An additional three cases from cluster B (Table 1), detected by the temporal scan statistic, were not detected by the space-time analysis (two cases in Albany and one in Erie Counties).

## Discussion

*L. monocytogenes* causes a rare, severe human foodborne disease and is responsible for an estimated 2,500 human cases and 500 deaths annually in the United States (*28*). Most of these cases have been considered sporadic, and comparatively few outbreaks have been reported worldwide (*1*). The best quantitative estimates of the true number of *L. monocytogenes* infections come from the FoodNet program, which conducts population-based active laboratory surveillance for foodborne diseases at 10 sites in the United States that represent 10.8% of the U.S. population (*2*). FoodNet aggregate data from 1996 to 2000 show that the reported listeriosis rates among participating sites ranged from 3 to 6 cases per million population per year. The 131 case isolates collected over 44 months in this study translate to a rate of 3.3 cases per million population per year. This rate is within the reported case prevalence from FoodNet sites (*2*), indicating that the capture rate achieved in this study is within the expected range.

### Comparison of Genotyping Methods

Rapid, reproducible, and discriminatory subtyping methods are important in conducting effective surveillance. While others have shown that PFGE and ribotyping are highly discriminatory for typing *L. monocytogenes* (*29*–*32*), no comprehensive reports evaluated typing strategies on human isolates from a broad-based surveillance program (*29*–*32*). While our data show that *Eco*RI ribotyping and *Asc*I PFGE typing provide discriminatory subtyping approaches for human listeriosis isolates (Simpson’s index of 0.923 and 0.975, respectively), we also found that most ribotypes could be further differentiated by *Asc*I PFGE. Since PFGE types of epidemiologically related isolates may differ by <3 bands from each other (*27*), small clusters (e.g., cluster D) involving related but distinct (<3 bands difference) isolates may not be detected by PFGE typing but can be detected by ribotyping, which appears to target more conserved genetic characteristics.

### Detection and Definition of Listeriosis Clusters and Outbreaks

Within the 3 1/2-year study period covered by this report, nine putative case clusters (representing 31% of cases) were identified by using the scan statistic based on ribotyping or PFGE data. Application of the scan statistic for cluster detection ensured that putative clusters accounted for the relative abundance of *L. monocytogenes* ribotypes and PFGE types. Five clusters, representing 18% of all cases, were supported statistically by both subtyping methods. Of the six ribotype clusters identified by using the scan statistic, all contained isolates with closely related PFGE types. When refined to include only closely related PFGE types (<3 bands difference), these six clusters represent 20% of the cases reported during the surveillance period. Cluster C contained five PFGE types, including three that were more than five bands different and two that were two bands different, indicating that these cases were unrelated. The relevance of both PFGE and ribotyping-based cluster detection by means of the scan statistic is supported by the observation that two of the clusters detected by one or both methods represent clusters that were part of epidemiologically confirmed multistate human foodborne listeriosis outbreaks. Cluster B (Table 1), which included 14 cases in New York State that were ribotype DUP-1044A, was part of a multistate outbreak with 101 cases (including 21 deaths) linked to eating *L. monocytogenes–*contaminated hot dogs (*12*,*33*). All cases from Cluster B, including the one case with a PFGE pattern that differed by a single band from the other isolates (Figure 3), were epidemiologically linked to the national outbreak. A second cluster (cluster G) was connected to cases in Maryland, New York, and Connecticut and was linked by subtyping and epidemiologically to contaminated paté (*34*,*35*). These two clusters represented 13% of all cases reported in New York State during this surveillance period. Because of the retrospective nature of this study, no epidemiologic data were available to link the cases representing the other subtype clusters.

While many reports claim that most listeriosis cases are sporadic (*2*–*4*,*7*), our data show that a considerable proportion of human listeriosis cases represent subtype clusters, some or all of which may represent common source outbreaks. Such clusters may have also occurred before 1997 and in other states and countries. While many of the subtype clusters detected in New York State appear to be small, some involved additional cases outside the state, and some cases connected with these clusters may never have been diagnosed. As nationwide surveillance and genotyping systems such as PulseNet (*36*) become fully implemented, a much larger number of human listeriosis clusters and outbreaks may be recognized and linked to specific food sources. Subtyping methods will only provide their full public health benefit if routine food histories are obtained for all listeriosis patients to provide the epidemiologic support for putative single genotype clusters. Complete routine food histories were not obtained as part of this study but were administered when putative outbreaks (such as clusters B and G) were detected before application of the statistical algorithm described here.

While some clusters defined by the temporal scan statistic also represented statistically significant spatial clusters (Figure 1, D, G, H, and I;), other temporal clusters included cases distributed across the state (A, B, C, E, and F). Cluster B included two smaller space-time clusters as well as other cases distributed across the state; all of these cases were epidemiologically linked. These patterns are consistent with those of previously reported human listeriosis outbreaks. Some previous outbreaks of listeriosis have been represented as geographic clusters associated with localized consumption of a contaminated food item (e.g., outbreaks in North Carolina [*37*] and California [*38*] linked to Hispanic-style cheeses). Other outbreaks were geographically dispersed and included cases in many states; these clusters were caused by a widely distributed contaminated food item (such as the multistate outbreak in 1998–99 [*12*]; cluster B). Our results further suggest that human listeriosis clusters and outbreaks may occur in two distinct patterns, including localized, geographically confined, and dispersed clusters. This epidemiologic spreading pattern indicates that time clustering is probably at least as effective in detecting clusters as combined space-time clustering.

### Cluster Detection Methods

While some efforts to track *L. monocytogenes* subtypes responsible for human cases over time have been published (*39*,*40*), we show that the use of comprehensive multimethod genotyping approaches in conjunction with formal statistical means for detecting putative listeriosis clusters may help provide a better understanding of the epidemiologic characteristics of this disease. While the combination of typing and normal distribution-based statistical algorithms for outbreak detection has been shown to be effective for detecting outbreaks for more common foodborne diseases such as salmonellosis (*41*), different approaches are needed to effectively detect clusters for rare diseases such as listeriosis. Therefore, we used PFGE and ribotyping subtyping in conjunction with the Poisson-distribution-based scan statistic to detect listeriosis clusters. The scan statistic was chosen since this method has previously been applied to detect clusters of other rare diseases, e.g., variant Creutzfeldt-Jakob disease (*24*,*42*,*43*). Because of the long incubation period of listeriosis, the scan statistic was performed by using both 1- and 3-month windows. Our data showed that all clusters detected with the 1-month window were also detected with the 3-month window size. Further validation of appropriate window sizes for these analyses by using epidemiologically confirmed outbreaks will be necessary to define the optimal parameters for the scan statistic analysis. While *Eco*RI ribotyping was shown to be less discriminatory than *Asc*I PFGE typing, PFGE patterns differing by <3 bands from each other may possibly be clonally related and share a recent enough common ancestor to be grouped together for epidemiologic investigations (*27*). Consequently, the use of the more discriminatory PFGE subtyping data alone may sometime miss clusters caused by clonally related isolates, which may not necessarily share completely identical PFGE patterns, if only the completely identical PFGE patterns (0 band difference) are grouped together as a single PFGE type. The use of only ribotyping data may overestimate the number of clusters because of the lower discriminatory ability of ribotyping. We showed that PFGE data further refined the initially defined ribotype clusters and eliminated clusters that contained isolates with distinct PFGE subtypes.

## Discussion

Conventional surveillance for listeriosis and other foodborne diseases often relies upon species or serotype characterization to define reportable conditions, yet for many organisms genotyping can provide improved discrimination below the species or serotype level. In conjunction with statistical analyses, routine genotyping allowed us to identify a considerable number of putative temporal clusters of listeriosis. Our data show that 13% of reported human listeriosis cases in New York State represented epidemiologically supported single-source, multicase clusters. On the basis of molecular subtyping data alone, as many as 31% of the listeriosis cases may have represented clusters. We propose that a considerable number of human listeriosis cases may occur in clusters, many or some of which may represent single-source outbreaks that in the past went undetected. The combined use of molecular subtyping methods, statistical data analysis, and epidemiologic investigations thus may further improve our ability to detect human listeriosis outbreaks.

The U.S. Department of Health and Human Services Healthy People 2010 plan calls for a reduction of human listeriosis from 0.5 to 0.25 cases per 100,000 by the year 2010 (*44*). Efforts to reduce *Listeria* species in the processing environment appear to have reduced the incidence of listeriosis from a peak of 0.8 cases per 100,000 in the early 1990s, but the incidence has remained at approximately 0.3–0.6 cases per 100,000 since 1996 (*7*,*45*). Our study suggests that single-source clusters represent a much larger number of listeriosis cases than previously assumed. We provide a model for an integrated, statistically based, molecular subtyping approach to identifying putative foodborne listeriosis clusters. In conjunction with broad-based collection of conventional epidemiologic data, this approach may allow for more rapid detection of even smaller outbreaks, which currently are often unrecognized. Rapid cluster detection can help detect and eliminate outbreak sources and prevent additional cases, thus providing an opportunity to reduce the overall incidence of foodborne listeriosis. Improved outbreak detection furthermore will provide an opportunity to better define the specific food sources of human listeriosis cases.
